# A lifestyle intervention improves sexual function of women with obesity and infertility: A 5 year follow-up of a RCT

**DOI:** 10.1371/journal.pone.0205934

**Published:** 2018-10-23

**Authors:** Vincent Wekker, Matty D. A. Karsten, Rebecca C. Painter, Cornelieke van de Beek, Henk Groen, Ben Willem J. Mol, Annemieke Hoek, Ellen Laan, Tessa J. Roseboom

**Affiliations:** 1 Department of Obstetrics and Gynecology, Amsterdam UMC, University of Amsterdam, Amsterdam, The Netherlands; 2 Amsterdam Reproduction and Development Research Institute, Amsterdam UMC, Amsterdam, The Netherlands; 3 Department of Clinical Epidemiology, Biostatistics and Bioinformatics, Amsterdam UMC, University of Amsterdam, Amsterdam, The Netherlands; 4 Amsterdam Public Health Research Institute, Amsterdam UMC, Amsterdam, The Netherlands; 5 Department of Obstetrics and Gynecology, University of Groningen, University Medical Centre Groningen, Groningen, The Netherlands; 6 Department of Epidemiology, University of Groningen, University Medical Centre Groningen, Groningen, The Netherlands; 7 Department of Obstetrics and Gynecology, Monash Medical Centre, Monash University, Clayton, Australia; 8 Department of Sexology and Psychosomatic Obstetrics and Gynecology, Amsterdam UMC, University of Amsterdam, Amsterdam, The Netherlands; University of Sydney, AUSTRALIA

## Abstract

**Background:**

Obesity and infertility are associated with poorer sexual function. We have previously shown that a lifestyle intervention in women with obesity and infertility reduced weight and improved cardiometabolic health and quality of life, which may positively affect sexual function. We now report on sexual function 5 years after randomization.

**Methods and findings:**

In total 577 women, between 18–39 years of age, with infertility and a BMI ≥29 kg/m^2^ were randomized to a six-month lifestyle intervention targeting physical activity, diet and behavior modification or prompt infertility care as usual. Intercourse frequency and sexual function were assessed with the McCoy Female Sexuality Questionnaire (MFSQ), 5.4±0.8 years after randomization. 550 women could be approached for the follow-up study, of whom 84 women in the intervention and 93 in the control group completed the MFSQ. Results were adjusted for duration of infertility, polycystic ovary syndrome and whether women were attempting to conceive. The intervention group more often reported having had intercourse in the past 4 weeks compared to the control group (aOR: 2.3 95% CI 0.96 to 5.72). Among women reporting intercourse in the past 4 weeks, the intervention group (n = 75) had intercourse more frequently (6.6±5.8 vs. 4.9±4.0 times; 95% CI 0.10 to 3.40) and had higher scores for vaginal lubrication (16.5±3.0 vs. 15.4±3.5; 95% CI 0.15 to 2.32) and total ‘sexual function’ score (96.5±14.2 vs. 91.4±12.8; 95% CI 0.84 to 9.35) compared to the control group (n = 72). Sexual interest, satisfaction, orgasm and sex partner scores did not differ statistically between the groups. The intervention effect on sexual function was for 21% mediated by the change in moderate to vigorous physical activity.

**Conclusion:**

A six-month lifestyle intervention in women with obesity and infertility led to more frequent intercourse, better vaginal lubrication and overall sexual function 5 years after the intervention. (Trial Registration: NTR1530).

## Introduction

Sexual function plays an important role in the quality of life of adults and is determined by both biological and psychosocial factors [1, 2]. The multi-dimensional nature of sexual function and the diversity in assessment methods make it difficult to estimate the prevalence of female sexual dysfunction, but estimates are up to 40% worldwide [3–5].

Obesity and sexual function are associated through various mental and physical pathways [6, 7]. In women, obesity leads to decreased fecundability and increases the demand for assisted reproductive techniques [8–10]. Infertile couples undergoing infertility treatment report a poorer sexual function suggesting a causal relationship between infertility and sexual function, however a reciprocal or bidirectional association has also been suggested [11–14]. Apart from this effect on reproduction, obesity increases the risks of cardiometabolic diseases that are also associated with lower sexual function, like type two diabetes, dyslipidemia and hypertension [15, 16]. Endothelial function and genital blood flow seem to form an important link between cardiometabolic health status and sexual function, although evidence on the cause-effect relationship is scarce [17–21]. In addition, there is conflicting evidence on whether metabolic syndrome increases the risk of sexual dysfunction [22, 23]. Anxiety and depression are more prevalent in the obese population, and are directly and indirectly linked to sexual function [24, 25]. Obesity may thus be a common etiological factor for decreased sexual function, explaining the co-occurrence of multiple conditions, including mental, metabolic, reproductive and sexual problems [11, 26].

The prevalence of obesity has been increasing and affects around 48% of all women of childbearing age in the United States [27]. Lifestyle interventions are the first step in the treatment of obesity and have shown to improve several domains of physical health, mental health and increase quality of life [28–34]. Weight-loss is associated with an increase in female sexual function, although not all intervention studies found such an effect [35]. Studies in obese and overweight women with and without type 2 diabetes showed better sexual function after lifestyle interventions, especially in women who had been diagnosed with a sexual dysfunction prior to the intervention [36, 37].

We have previously reported on the LIFEstyle study, a Randomized Controlled Trial (RCT) in which women with obesity and infertility were allocated to a six month lifestyle intervention or infertility care as usual [38]. This intervention led to weight loss, improved cardiometabolic health by halving the odds of metabolic syndrome and improved physical quality of life [30, 39]. Due to these effects that are associated with sexual function, we hypothesized that a lifestyle intervention in women with obesity and infertility would improve sexual function. The current follow-up study of the LIFEstyle study aimed to investigate the effects of a lifestyle intervention in women with obesity and infertility on sexual function.

## Methods

The current study is a follow-up study of the women who participated in the LIFEstyle study, a multicenter RCT. The study was conducted according to the principles of the Declaration of Helsinki and approved by the medical ethics committee of the University Medical Centre Groningen (UMCG) (METc code: 2008/284), as well as by the board of directors of the 22 other participating hospitals (Dutch trial register (NTR 1530)).

### LIFEstyle study

The original LIFEstyle study was conducted in 23 medical centers in the Netherlands. The protocols of the original LIFEstyle study and the current follow-up study (WOMB project) have been published previously [38, 40]. From June 2009 until June 2012, 577 women between 18–39 years of age with infertility and a Body Mass Index (BMI) ≥29 kg/m^2^ were randomly allocated (1:1) to a lifestyle intervention or infertility care as usual. Infertility was defined as chronic anovulation [41] or unsuccessful conception for at least 12 months [42]. Women with severe endometriosis, premature ovarian insufficiency, endocrinopathy (e.g. diabetes type I, Cushing’s syndrome), and untreated pre-conception hypertension, or hypertension-related complications in a previous pregnancy were not eligible, as were women treated with donor sperm. Randomization was performed with an online program at the Academic Medical Centre in Amsterdam, stratified for trial center and ovulatory status.

### Lifestyle intervention

Women allocated to the intervention group received a six-month structured lifestyle intervention, prior to receiving infertility treatment. The lifestyle intervention consisted of six face-to-face consultations of approximately 30 minutes at the outpatient clinics and four consultations by telephone and/or e-mail with trained research nurses. The goal of the lifestyle intervention was a 5–10% weight reduction, or a reduction in BMI below 29 kg/m^2^ within the intervention period. Women who reached this goal could stop with the lifestyle intervention program and proceed with infertility treatment. The intervention was discontinued if pregnancy occurred, but women could re-enter the intervention in case of a miscarriage.

The intervention was based on recommendations of the National Institute of Health [43], and consisted of a dietary, physical activity and a behavioral modification component. Women were advised to reduce their daily caloric intake by 600 kcal, but not below 1200 kcal/day. An online food diary was used to provide feedback on their diet. Women were recommended to be more physical active and to increase their daily step count to a minimum of 10,000 steps per day, supported by a pedometer that was worn daily. In addition, women had to be moderately physically active two to three times a week for a minimum of 30 minutes. Individual motivational counselling was used to set individual goals and create awareness of lifestyle factors predisposing to obesity [44]. Women were advised to adhere to their healthier lifestyle, also after finishing the intervention.

### Control strategy

Women in the control group were treated according to the Dutch infertility guidelines, irrespective of their BMI, and were given information about the negative effects of obesity on fertility, as part of the usual care in the Netherlands [42].

### Study procedures

All women who participated in the LIFEstyle study were eligible for the current follow-up study, five years after randomization. All women for whom valid contact information was available were sent an invitation letter in which they received information about the follow-up study. Women were contacted by telephone if they did not respond to the letter. All participants provided written informed consent. Participants could fill out a paper or online version of the questionnaires at home, without the presence of a researcher.

### Outcome measures

Women filled out questionnaires concerning demographics, current lifestyle, reproductive health, quality of life (36-Item Short Form Survey), and anthropometrics [45]. The amount of moderate to vigorous physical activity (MVPA) in minutes per week was assessed with the validated Short QUestionnaire to ASsess Health-enhancing physical activity (SQUASH), which collects information about commuting activities, leisure time activities, household activities, activities at work and school, using three main questions: days per week, average time per day/week, and intensity [46].

The main outcomes of this paper were assessed using the Dutch version of the validated McCoy Female Sexuality Questionnaire (MFSQ) [47]. The MFSQ has a good test-retest reliability (*r* = 0.71–0.95) and discriminating capacity between women with and without sexual dysfunction. [48] In this 19-item questionnaire, 18 items are scored on a 7-point Likert scale. The remaining item asks about frequency of intercourse in the past 4 weeks. The questionnaire investigates five dimensions of sexual health: sexual interest; satisfaction; vaginal lubrication; orgasm and sex partner. Intercourse frequency, as part of the sexual satisfaction domain, is converted into a 7-point scale on a percentage-wise basis, in which the lowest frequency is converted to 1 point and the highest to 7 points. To calculate domain scores, ‘non applicable’ answers were replaced by the mean of at least 2 of the other items of the corresponding domain. The total score is calculated in complete cases by the sum score of all individual items. Only women who reported intercourse could complete all of the 19 items [47].

### Statistical analyses

Comparison of baseline and follow-up characteristics was performed based on treatment group. We assessed potential selection bias by comparing the baseline characteristics between participants and non-participants. We analysed continuous variables using an independent sample t-test, binary and categorical outcomes with a Pearson Chi-Square, Fisher’s exact test or Fisher-Freeman-Halton exact test; p-values <0.05 were considered statistically significant. Sexual activity was assessed in the complete sample of women who filled out the questionnaire. In line with the MFSQ user manual, the frequency of intercourse, the five domains concerning sexual function and total MFSQ score were analysed for all women who reported having had intercourse at least once during the past four consecutive weeks [47]. We analysed the differences in outcomes between the intervention and control group by logistic and linear regression analyses. Duration of infertility at baseline, polycystic ovary syndrome (PCOS) and attempting to conceive (yes or no) were added as covariates to the adjusted model. PCOS was diagnosed by the Rotterdam 2003 criteria [49]. Results are presented as odds ratios for sexual activity or mean difference in intercourse frequency, domain scores or total MFSQ score between the intervention and control group. Confidence intervals (CI) for continuous outcomes are reported as bias corrected and accelerated (BCa) 95% CI, based on 5000 bootstrap samples [50]. Confidence intervals not including zero were considered statistically significant. Post-hoc mediation analyses were performed for the total score of the MFSQ, including delta values between baseline and follow-up of factors attributable to the intervention: weight, waist- and hip circumference, mental and physical quality of life and MVPA. The mediating effects were analysed for all potential mediators separately and combined. The mediation analyses were performed using model 4, with 5000 bootstrapped samples for the estimation of bias corrected 95% CI, of the PROCESS macro (V.2.16.3) for SPSS [51]. All statistical analyses were performed using IBM SPSS version 24.0 (Armonk, NY, USA). At the start of the original trial, no power calculation was performed for sexual function as a long-term outcome [38].

## Results

### Participation

#### Flow of participants

**Fig 1:**
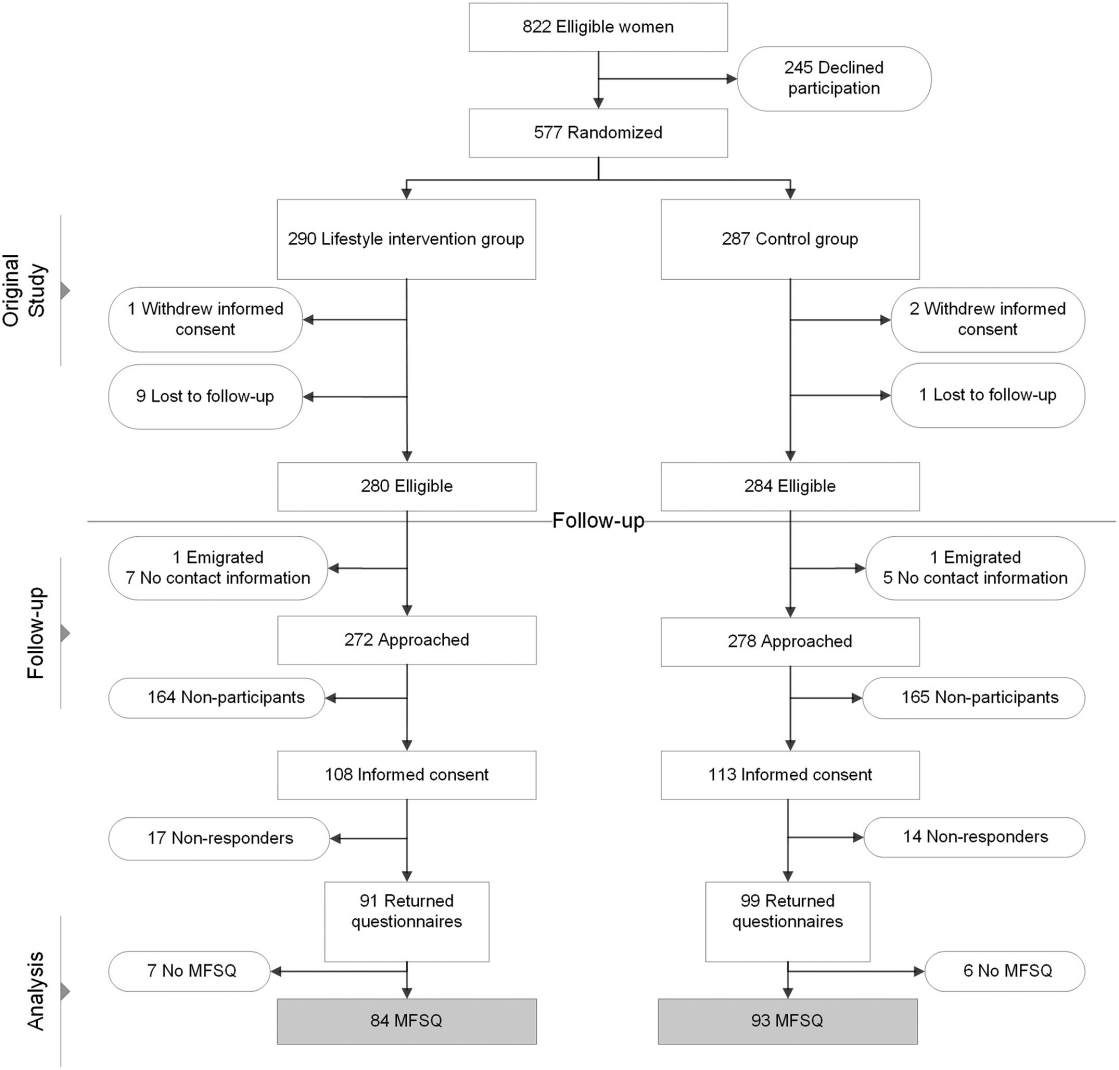
Flowchart of participants. **Abbreviations**: MFSQ, McCoy Female Sexuality Questionnaire.

During the original trial, 577 women were randomized, of which 3 women withdrew their informed consent and 10 women were lost to follow-up. Of the 564 women who completed the original trial, 14 women could not be contacted for the current follow-up study, because of missing contact information or immigration out of the Netherlands. All remaining 550 women (98%) were approached for the follow-up study, of whom 272 women in the intervention group and 278 women in the control group. A total of 106 of the approached women (39%) in the intervention and 113 women (41%) in the control group gave written informed consent. 31 of the women who gave informed consent did not respond to the complete set of questionnaires, and 13 women did not fill out the MFSQ specifically, because of personal reasons (not specified). In total, 84 of the approached women (31%) in the intervention group and 93 women (33%) in the control group filled out the MFSQ and were included in the analyses (Fig 1). Of the women in the intervention group, 13 women (15,5%) did not complete the intervention program.

#### Participants and non-participants

The comparison of women who filled out the MFSQ (N = 177) and women who did not (N = 397) is shown in S2 Table. Women who filled out the MFSQ were more often Caucasian, were more often diagnosed with PCOS, but had shorter duration of infertility and a higher score for mental quality of life at baseline than women who did not fill out the MFSQ.

#### Characteristics of treatment groups

The characteristics of the women who participated in the follow-up are reported separately for the intervention and control group in Tables 1 and 2 respectively. Women in the intervention group had a longer duration of infertility at baseline (Table 1).

**Table 1:** Baseline characteristics of the participants.

Variables	n	Intervention group	n	Control group	P-value ^a^
Age, years–mean (SD)	84	30.2 (4.1)	93	29.7 (4.3)	0.40
Weight, kg–mean (SD)	84	104.8 (12.8)	93	103.6 (11.9)	0.52
Waist circumference, cm mean–(SD)	80	107.6 (9.6)	93	108.7 (9.4)	0.42
Hip circumference, cm mean–(SD)	82	124.7 (8.7)	93	125.1 (8.4)	0.76
Caucasian–no. (%)	84	79 (94.0)	93	90 (96.8)	0.48
Education–no. (%)	81		91		0.66
Primary school, age 4–12 year		3 (3.7)		1 (1.1)	
Secondary education		15 (18.5)		20 (22.0)	
Intermediate vocational education		44 (54.3)		51 (56.0)	
Advanced vocational education or university		19 (23.5)		19 (20.9)	
Current smoker–no. (%)	83	20 (24.1)	92	17 (18.5)	0.36
Nulliparous–no. (%)	84	65 (77.4)	93	68 (73.1)	0.51
Duration of infertility–median (IQR)	84	20.5 (14.0–37.0)	93	17.0 (12.0–24.5)	0.04
Polycystic Ovary Syndrome ^b^—no. (%)	84	31 (36.9)	93	42 (45.2)	0.27
Physical Quality of Life–median (IQR)	68	53.0 (47.6–55.4)	83	51.3 (45.6–54.4)	0.12
Mental Quality of Life–median (IQR)	68	53.8 (50.1–57.1)	83	53.8 (48.7–56.2)	0.42
Weekly intercourse frequency, median (IQR)	65	3.0 (2.0–3.0)	81	2.0 (2.0–3.0)	0.85

^a^ P-values of continuous outcomes based on student t-test or Mann-Whitney-U test. P-values of dichotomous and categorical outcomes are based on the Pearson Chi-Square test, the Fisher’s exact test or Fisher-Freeman-Halton exact test.

^b^ Diagnosed by Rotterdam 2003 criteria [49].

**Abbreviations**: n, number; SD, Standard Deviation.

**Table 2:** Follow-up characteristics of the participants.

Variables	n	Intervention group	n	Control group	P-value ^a^
Age at follow-up, years–mean (SD)	84	35.6 (4.3)	93	35.2 (4.4)	0.49
Follow-up duration, years–mean (SD)	84	5.4 (0.9)	93	5.5 (0.7)	0.55
Weight, kg–mean (SD)	84	99.6 (15.1)	93	99.8 (16.5)	0.95
Waist circumference, cm mean–(SD)	82	107.3 (13.5)	92	108.3 (13.3)	0.62
Hip circumference, cm mean–(SD)	82	120.0 (11.4)	92	120.5 (14.4)	0.79
Long-term relationship ^b^ –no. (%)	84	75 (89.3)	93	87 (93.5)	0.42
Childlessness–no. (%)	84	18 (21.4)	93	14 (15.1)	0.27
History of miscarriage ^c^—no. (%)	84	28 (33.3)	93	31 (33.3)	1.00
Attempting to conceive—no. (%)	84	23 (27.4)	93	15 (16.1)	0.07

^a^ P-values of continuous outcomes based on student t-test or Mann-Whitney-U test. P-values of dichotomous and categorical outcomes are based on the Pearson Chi-Square test or the Fisher’s exact test.

^b^ Women who are in a relationship with the same partner as during the intervention.

^c^ including three women with a history of extra uterine gravidity.

**Abbreviations**: n, number; SD, Standard Deviation.

### Sexual intercourse occurrence

Of the 177 women who filled out the MFSQ, 75 of the 84 women (89.3%) in the intervention group compared to 72 of the 93 women (77.4%) in the control group reported having had intercourse in the past four weeks, resulting in an Odds Ratio (OR) of 2.4 (95% CI 1.04–5.66; p = 0.04). However, the OR was not statistically significant after adjusting for duration of infertility at baseline, PCOS and whether women were attempting to conceive (aOR: 2.3 95% CI 0.96–5.72; p = 0.06). (Table 2).

In the group of women who reported having had intercourse (irrespective of the treatment group), the prevalence of PCOS (44.2% versus 26.7%; p = 0.08) as well as the percentage of women attempting to conceive (24.5% versus 6.7% p = 0.03) was higher in comparison to women who reported not to have had intercourse. Furthermore, women who reported not to have had intercourse had a lower frequency of intercourse at baseline compared to women who had intercourse at follow-up (median 2.0 (IQR 1.0–3.0) versus 3.0 (2.0–3.0) per week; p = 0.03) (S3 Table).

### Intercourse frequency and sexual function

#### Baseline- and follow-up characteristics of women reporting intercourse

The between group comparison of characteristics of women who reported having had intercourse in the last four weeks, at baseline and follow-up is reported in S4 Table. Women in the intervention group had a longer duration of infertility at baseline (20.0 (IQR 14.0–40.0) versus 17.0 (12.0–23.8) months; p = 0.04).

#### Intervention effect on intercourse frequency and sexual function

Among women reporting intercourse, the frequency of intercourse was higher in the intervention group than in the control group (Table 3). Women in the intervention group also had higher total MFSQ scores and higher scores on the sexual satisfaction and vaginal lubrication domains (Table 3). After adjusting for duration of infertility at randomization, PCOS and whether women were attempting to conceive at time of the outcome assessment, the difference in sexual satisfaction scores was not statistically significant. Sexual interest, orgasm and sex partner domain scores were higher in the intervention group, but were not statistically significant (Table 3).

**Table 3:** Comparison of intercourse frequency, MFSQ domains and total score in women reporting intercourse in the past four weeks.

Outcomes			Unadjusted		Adjusted ^a^	
	Intervention group (n = 75)	Control group (n = 72)	Mean difference	95% CI ^b^	Mean difference	95% CI ^b^
Intercourse frequency, number per 4 weeks–mean (SD)	6.6 (5.8)	4.9 (4.0)	1.7	0.18–3.25	1.7	0.10–3.40
Sexual interest, score–mean (SD)	28.1 (6.2)	26.3 (5.8)	1.9	-0.11–3.83	1.8	-0.07–3.67
Sexual satisfaction, score–mean (SD)	11.8 (2.6)	10.9 (2.6)	0.9	0.07–1.75	0.9	-0.03–1.73
Vaginal lubrication, score–mean (SD)	16.5 (3.0)	15.4 (3.5)	1.1	0.07–2.21	1.3	0.15–2.32
Orgasm, score–mean (SD)	20.8 (5.0)	19.5 (5.2)	1.2	-0.41–2.85	1.3	-0.27–2.81
Sex partner, score–mean (SD)	19.3 (1.9)	19.0 (2.0)	0.2	-0.39–0.86	0.2	-0.43–0.87
Total MFSQ, score–mean (SD)	96.5 (14.2)	91.4 (12.8)	5.1	0.87–9.48	5.1	0.84–9.35

^a^ Adjusted for duration of infertility at randomization, PCOS and whether women were attempting to conceive at time of the outcome assessment.

^b^ Bias corrected and accelerated 95% CIs based on 5000 bootstrap re-samples; CI not containing zero indicate statistical significance.

**Abbreviations**: n, number; SD, Standard Deviation; CI, Confidence Interval; MFSQ, McCoy Female Sexuality Questionnaire.

#### Mediation of the lifestyle intervention effect on sexual function

The mediation analyses showed that 21% of the total intervention effect on the total MFSQ score at time of follow-up was mediated by MVPA. No statistically significant mediating effects of change in weight, change in waste- and hip circumference, or change in mental or physical quality of life were observed on the intervention effect on the total MFSQ score. The combination of the mediators into one model explained 37% of the total intervention effect on MFSQ total score (Table 4).

**Table 4:** Mediation of change in measures of anthropometrics, physical activity and quality of life on MFSQ total score.

Mediator	n	Indirect effect, (95% CI) ^a^	Mediation effect, % (95% CI) ^b^
Δ Weight, kg	146	0.10 (-0.26–1.11)	1.9% (-5.1–21.6)
Δ Waist circumference, cm	140	-0.00 (-0.46–0.48)	0.0% (-9.7–10.2)
Δ Hip circumference, cm	142	0.17 (-0.21–1.33)	3.8% (-4.6–28.8)
Δ Mental quality of life score	118	-0.02 (-0.77–0.34)	-0.4% (-13.8–6.0)
Δ Physical quality of life score	118	0.17 (-0.58–1.92)	3.1% (-10.3–34.1)
Δ MVPA, minutes/week ^c^	129	1.05 (0.13–2.90)	20.7% (2.6–56.9)
Combined model ^d^	113	1.91 (0.14–4.47)	36.9% (2.8–86.3)

^a^ Bias corrected 95% CIs based on 5000 bootstrap re-samples. CI not containing zero indicate statistical significance.

^b^ Mediation effect is indirect effect / total effect x 100%. CI not containing zero indicate statistical significance.

^c^ MVPA = Moderate to vigorous physical activity in minutes per week based on SQUASH questionnaire.

^d^ Model includes Δ weight, Δ waist circumference, Δ hip circumference, Δ mental quality of life score, Δ physical quality of life score, Δ MVPA between baseline and follow-up as mediator variables.

**Abbreviations**: n, Number; BC, Bias Corrected; CI, Confidence Interval.

## Discussion

This five-years follow-up study of an RCT shows that a lifestyle intervention in women with obesity and infertility improves sexual function. Five years after the intervention, women in the intervention group reported a higher intercourse frequency, more vaginal lubrication and a better overall sexual function than women in the control group. The intervention group also scored higher on the sexual interest, satisfaction, orgasm and sex partner domains, but these effects were not statistically significant. Our finding that the lifestyle intervention did not only reduced weight, improved cardiovascular health and physical quality of life in the short term, but also had lasting beneficial effects on sexual function shows that lifestyle interventions have beneficial effects for health in a broad range of areas of health and wellbeing [39].

Randomized studies of lifestyle interventions in women with other comorbidities like type two diabetes, that were not specifically performed in women with obesity and infertility reported positive as well as absent effects on sexual function during or directly after a lifestyle intervention [35, 37, 52]. A non-randomized study with a follow-up period of 2 years reported improved sexual quality of life after a lifestyle intervention that successfully reduced weight among obese women. Regaining body weight did not eliminate the increase in sexual quality of life 2 years later [53]. We found similar results showing better sexual function in the intervention group, despite the absence of a difference in weight at follow-up. This suggest that a lifestyle intervention can have beneficial long-term effects on sexual health, even in the absence of a long-term effect on weight.

The intervention effect on sexual function was partly mediated by change in physical activity. In our study, a decrease in physical activity was associated with a lower sexual function (*R* = 0.23; p <0.01). Although throughout the study, physical activity decreased over time, the intervention group reduced their physical activity less than the control group (mean delta: -111.4 vs. -492 min/week; p = 0.03), which seems to have contributed to a better sexual function in the intervention group compared to the control group.

This study is the first to report experimental evidence of better long-term sexual function in women with obesity and infertility after a lifestyle intervention. The validated MFSQ was part of a more extensive survey that was filled out in private, without the presence of a researcher, which probably has reduced the risks of performance bias and social desirability bias, increasing the reliability of our results [54].

We were unable to assess the change in sexual function over time, because sexual function was not assessed at the start of the study. Whether the difference between the intervention and control group was based on an improved sexual function in the intervention group or decreased sexual function in the control group could not be determined. Adjustments for duration of infertility at randomization, PCOS, and attempting to conceive had little effect on our results, therefore it seems unlikely that our finding of a better sexual function in the intervention group was caused by these potential confounders. However, we found indications for selective attrition by comparing the 177 participants (31.4%) with the non-participants. Women who participated in the follow-up were more often Caucasian, were more often diagnosed with PCOS, but had a shorter duration of infertility and higher mental quality of life at start of the intervention. PCOS has been associated with a poorer sexual health, whereas shorter infertility duration and higher mental quality of life have been associated with a better sexual function [12, 34, 55]. Due to these opposed associations with sexual function and small absolute differences it seems unlikely that the selection in participants (S2 Table) had a substantial impact on our findings. However, the high percentage (>95%) of Caucasian women in our sample does limit the representativeness of our findings to the Caucasian population. Whether similar effects can be found in non-Caucasian women needs to be further investigated.

Our study shows that even in the absence of a sustained effect on weight, a lifestyle intervention in women with obesity and infertility leads to more frequent intercourse, better vaginal lubrication and overall sexual function 5 years later. Thus besides short-term improvements in cardiometabolic health and quality of life, lifestyle interventions can contribute to a better long-term sexual function in women who are at greater risk of sexual problems.

## Supporting information

S1 File Research protocol as approved by the ethics committee. (PDF)

S2 File Minimal data set. (SAV)

S1 Table CONSORT checklist. (PDF)

S2 Table Comparison of baseline characteristics of participants and non-participants. (DOCX)

S3 Table Comparison of women who had intercourse and women who did not had intercourse in past four weeks. (DOCX)

S4 Table Baseline and follow-up characteristics of all women who reported intercourse in past four weeks. (DOCX)
