# Supplementary material for: A lifestyle intervention improves sexual function of women with obesity and infertility: A 5 year follow-up of a RCT
Source: PLoS One. 2018 Oct 23;13(10):e0205934. doi: 10.1371/journal.pone.0205934 (PMC6198949; doi:10.1371/journal.pone.0205934)
Supplement: S2 Table — (DOCX) [file pone.0205934.s004.docx]

**S2 Table. Comparison of baseline characteristics of participants and non-participants.**

| **Variables** | **n** | **Non-participants** | **n** | **Participants** | **P-value ^a^** |
| --- | --- | --- | --- | --- | --- |
| Age, years – mean (SD) | 397 | 29.7 (4.7) | 177 | 30.0 (4.2) | 0.45 |
| Weight, kg – mean (SD) | 395 | 102.8 (13.3) | 177 | 104.2 (12.3) | 0.26 |
| Waist circumference, cm mean – (SD) | 391 | 107.9 (9.3) | 173 | 108.2 (9.4) | 0.75 |
| Hip circumference, cm mean – (SD) | 391 | 125.1 (9.0) | 175 | 125.0 (8.5) | 0.84 |
| Caucasian – no. (%) | 397 | 333 (83.9) | 177 | 169 (95.5) | <0.01 |
| Education – no. (%) | 377 |  | 172 |  | 0.07 |
| Primary school, age 4-12 year |  | 23 (6.1) |  | 4 (2.3) |  |
| Secondary education |  | 96 (25.5) |  | 35 (20.3) |  |
| Intermediate vocational education |  | 171 (45.4) |  | 95 (55.2) |  |
| Advanced vocational education or university |  | 87 (23.1) |  | 38 (22.1) |  |
| Current smoker – no. (%) | 394 | 99 (25.1) | 175 | 37 (21.1) | 0.30 |
| Nulliparous – no. (%) | 396 | 308 (77.8) | 177 | 133 (75.1) | 0.49 |
| Duration of infertility – median (IQR) | 394 | 22.0 (14.0 – 36.0) | 177 | 19.0 (13.0 – 30.0) | 0.03 |
| Polycystic Ovary Syndrome ^b^ - no. (%) | 395 | 128 (32.4) | 177 | 73 (41.2) | 0.04 |
| Physical Quality of Life – median (IQR) | 312 | 52.8 (47.8 – 55.9) | 151 | 52.1 (46.4 – 55.3) | 0.19 |
| Mental Quality of Life – median (IQR) | 312 | 52.0 (42.7 – 55.7) | 151 | 53.8 (49.3 – 56.8) | <0.01 |
| Weekly intercourse frequency, median (IQR) | 290 | 2.0 (2.0 – 3.0) | 146 | 2.0 (2.0 – 3.0) | 0.38 |

^a^ P-values of continues outcomes based on student t-test or Mann-Whitney-U test. P-values of dichotomous and categorical outcomes are based on the Pearson Chi-Square test, the Fisher’s exact test or Fisher-Freeman-Halton exact test.

^b^ Diagnosed by Rotterdam 2003 criteria [37].

**Abbreviations:** n, number; SD, Standard Deviation.
