# Supplementary material for: A lifestyle intervention improves sexual function of women with obesity and infertility: A 5 year follow-up of a RCT
Source: PLoS One. 2018 Oct 23;13(10):e0205934. doi: 10.1371/journal.pone.0205934 (PMC6198949; doi:10.1371/journal.pone.0205934)
Supplement: S3 Table — (DOCX) [file pone.0205934.s005.docx]

**S3 Table. Comparison of women who had intercourse and women who did not had intercourse in past four weeks.**

| **Variables** | **n** | **No Intercourse** | **n** | **Intercourse** | **P value ^a^** |
| --- | --- | --- | --- | --- | --- |
| Baseline Characteristics | | | | | |
| Age, years – mean (SD) | 30 | 30.5 (4.1) | 147 | 29.8 (4.3) | 0.41 |
| Weight, kg – mean (SD) | 30 | 102.9 (13.3) | 147 | 104.4 (12.2) | 0.53 |
| Waist circumference, cm mean – (SD) | 30 | 108.6 (10.6) | 143 | 108.1 (9.2) | 0.81 |
| Hip circumference, cm mean – (SD) | 30 | 124.6 (9.7) | 145 | 125.0 (8.3) | 0.80 |
| Caucasian – no. (%) | 30 | 29 (96.7) | 147 | 140 (95.2) | 1.0 |
| Education – no. (%) | 28 |  | 144 |  | 0.98 |
| Primary school, age 4-12 year |  | 0 (0) |  | 4 (2.8) |  |
| Secondary education |  | 5 (17.9) |  | 30 (20.8) |  |
| Intermediate vocational education |  | 17 (60.7) |  | 78 (54.2) |  |
| Advanced vocational education or university |  | 6 (21.4) |  | 32 (22.2) |  |
| Current smoker – no. (%) | 29 | 5 (17.2) | 146 | 32 (21.9) | 0.57 |
| Nulliparous – no. (%) | 30 | 24 (80.0) | 147 | 109 (74.1) | 0.50 |
| Duration of infertility – median (IQR) | 30 | 19.5 (13.8 – 29.3) | 147 | 19.0 (12.0 – 30.0) | 0.71 |
| Polycystic Ovary Syndrome ^b^ - no. (%) | 30 | 8 (26.7) | 147 | 65 (44.2) | 0.08 |
| Physical Quality of Life – median (IQR) | 29 | 52.1 (49.1 – 55.7) | 122 | 52.1 (45.8 – 54.9) | 0.48 |
| Mental Quality of Life – median (IQR) | 29 | 54.1 (49.0 – 57.2) | 122 | 53.3 (49.3 – 56.3) | 0.92 |
| Weekly intercourse frequency, median (IQR) | 28 | 2.0 (1.0 – 3.0) | 118 | 3.0 (2.0 – 3.0) | 0.03 |
| *Follow-up characteristics* | | | | | |
| Age at follow-up, years – mean (SD) | 30 | 36.1 (4.1) | 147 | 35.3 (4.4) | 0.35 |
| Follow-up duration, months – mean (SD) | 30 | 5.5 (0.9) | 147 | 5.4 (0.8) | 0.52 |
| Weight, kg – mean (SD) | 30 | 97.5 (15.6) | 147 | 100.2 (15.9) | 0.41 |
| Waist circumference, cm mean – (SD) | 29 | 107.5 (15.1) | 145 | 107.8 (13.1) | 0.90 |
| Hip circumference, cm mean – (SD) | 29 | 118.7 (15.8) | 145 | 120.6 (12.4) | 0.47 |
| No child - no. (%) | 30 | 5 (16.7) | 147 | 27 (18.4) | 0.83 |
| Attempting to conceive - no. (%) | 30 | 2 (6.7) | 147 | 36 (24.5) | 0.03 |

^a^ P-values of continues outcomes based on student t-test or Mann-Whitney-U test. P-values of dichotomous and categorical outcomes are based on the Pearson Chi-Square test, the Fisher’s exact test or Fisher-Freeman-Halton exact test.

^b^ Diagnosed by Rotterdam 2003 criteria [37].

**Abbreviations:** n, number; SD, Standard Deviation.
