# Supplementary material for: A lifestyle intervention improves sexual function of women with obesity and infertility: A 5 year follow-up of a RCT
Source: PLoS One. 2018 Oct 23;13(10):e0205934. doi: 10.1371/journal.pone.0205934 (PMC6198949; doi:10.1371/journal.pone.0205934)
Supplement: S4 Table — (DOCX) [file pone.0205934.s006.docx]

**S4 Table. Baseline and follow-up characteristics of all women who reported intercourse in past four weeks.**

| **Variable** | **n** | **Intervention group** | **n** | **Control group** | **P value** |
| --- | --- | --- | --- | --- | --- |
| *Baseline characteristics* | | | | | |
| Age, years – mean (SD) | 75 | 30.1 (4.1) | 72 | 29.6 (4.5) | 0.48 |
| Weight, kg – mean (SD) | 75 | 105.1 (11.8) | 72 | 103.7 (12.6) | 0.50 |
| Waist circumference, cm mean – (SD) | 71 | 107.7 (9.1) | 72 | 108.5 (9.4) | 0.58 |
| Hip circumference, cm mean – (SD) | 73 | 125.0 (7.8) | 72 | 125.1 (8.8) | 0.94 |
| Caucasian – no. (%) | 75 | 71 (94.7) | 72 | 69 (95.8) | 1.0 |
| Education – no. (%) | 74 |  | 70 |  | 0.71 |
| Primary school, age 4-12 year |  | 3 (4.1) |  | 1 (1.4) |  |
| Secondary education |  | 14 (18.9) |  | 16 (22.9) |  |
| Intermediate vocational education |  | 39 (52.7) |  | 39 (55.7) |  |
| Advanced vocational education or university |  | 18 (24.3) |  | 14 (20.0) |  |
| Current smoker – no. (%) | 75 | 19 (25.3) | 71 | 13 (18.3) | 0.31 |
| Nulliparous – no. (%) | 75 | 58 (77.3) | 72 | 51 (70.8) | 0.37 |
| Duration of infertility – median (IQR) | 75 | 20.0 (14.0 – 40.0) | 72 | 17.0 (12.0 – 23.8) | 0.04 |
| Polycystic Ovary Syndrome ^b^ - no. (%) | 75 | 29 (38.7) | 72 | 36 (50.0) | 0.17 |
| Physical Quality of Life – median (IQR) | 59 | 52.8 (46.7 – 55.0) | 63 | 51.3 (45.6 – 54.4) | 0.28 |
| Mental Quality of Life – median (IQR) | 59 | 53.2 (50.1 – 56.8) | 63 | 54.0 (48.6 – 56.2) | 0.64 |
| Weekly intercourse frequency, median (IQR) | 56 | 3.0 (2.0 – 3.0) | 62 | 3.0 (2.0 – 3.0) | 0.73 |
| *Follow-up characteristics* | | | | | |
| Age at follow-up, years – mean (SD) | 75 | 35.5 (4.3) | 72 | 35.0 (4.6) | 0.56 |
| Follow-up duration, years – mean (SD) | 75 | 5.4 (0.9) | 72 | 5.5 (0.7) | 0.64 |
| Weight, kg – mean (SD) | 75 | 99.8 (15.3) | 72 | 100.5 (16.6) | 0.79 |
| Waist circumference, cm mean – (SD) | 73 | 107.4 (14.0) | 72 | 108.3 (12.2) | 0.65 |
| Hip circumference, cm mean – (SD) | 73 | 119.7 (11.6) | 72 | 121.5 (13.2) | 0.39 |
| Childlessness - no. (%) | 75 | 15 (20.0) | 72 | 12 (16.7) | 0.60 |
| Attempting to conceive - no. (%) | 75 | 23 (30.7) | 72 | 13 (18.1) | 0.08 |

^a^ P-values of continues outcomes based on student t-test or Mann-Whitney-U test. P-values of dichotomous and categorical outcomes are based on the Pearson Chi-Square test, the Fisher’s exact test or Fisher-Freeman-Halton exact test.

^b^ Diagnosed by Rotterdam 2003 criteria [37].

**Abbreviations:** n, number; SD, Standard Deviation.
